# Revealing the structural microenvironment of high metastatic risk uveal melanomas following decellularisation

**DOI:** 10.1038/s41598-024-78171-2

**Published:** 2024-11-05

**Authors:** Karen Aughton, Joshua Hattersley, Sarah E Coupland, Helen Kalirai

**Affiliations:** 1 Liverpool Ocular Oncology Research Group, Department of Eye and Vision Science, Institute of Life Course and Medical Science, University of Liverpool, 3rd Floor William Henry Duncan Building, West Derby Street, Liverpool, L7 8TX UK; 2 Liverpool Clinical Laboratories, Liverpool University Hospital Foundation Trust, Liverpool, UK

**Keywords:** Uveal melanoma, Tumour microenvironment, Extracellular matrix, Proteomics, Metastatic risk, Decellularisation, Cancer microenvironment, Proteomics, Electron microscopy, Cellular signalling networks

## Abstract

Uveal melanoma (UM) is a rare aggressive intraocular tumour that spreads most commonly to the liver in tumours with loss of one copy of chromosome 3 (HR-M3); current treatments for metastatic disease remain largely ineffective. Pre-clinical research is increasingly using three-dimensional models that better recapitulate the tumour microenvironment (TME). One aspect of the TME is the acellular extracellular matrix (ECM) that influences cell proliferation, migration and response to therapy. Although commercial matrices are used in culture, the composition and biochemical properties may not be representative of the tumour ECM in vivo. This study identifies UM metastatic risk specific ECM proteins by developing methodology for decellularisation of low- and high- metastatic risk tissue samples (LR-D3 vs. HR-M3). Proteomic analysis revealed a matrisome signature of 34 core ECM and ECM-associated proteins upregulated in HR-M3 UM. Combining additional UM secretome and whole cell iTRAQ proteomic datasets revealed enriched GO and KEGG pathways including ‘regulating ECM binding’ and ‘PI3K/Akt signalling’. Structural analyses of decellularised matrices revealed microarchitecture of differing fibre density and expression differences in collagen 4, collagen 6A1 and nidogen 1, between metastatic risk groups. This approach is a powerful tool for the generation of ECM matrices relevant to high metastatic risk UM.

**Supplementary Information:**

The online version contains supplementary material available at 10.1038/s41598-024-78171-2.

## Introduction

Uveal melanoma (UM) is a rare aggressive intraocular tumour that accounts for around 13% of all melanoma deaths^1^. Although the primary tumour (pUM) is usually effectively treated, it metastasises in approximately 50% of patients, most commonly to the liver^2,3^. Metastatic UM (mUM) has limited treatment options and overall survival is short^4^. The treatment of mUM is a significant challenge due to diffuse dissemination of UM cells in the liver, and a lack of effective systemic treatments, including immunotherapy, in most affected patients.

The tumour microenvironment (TME) plays a critical role in tumour growth and the development of metastasis where the interaction between tumour cells and the associated stroma and cellular components, modulate tumour progression and patient prognosis^5,6^. We have previously demonstrated that the secretome of pUM with a high risk (HR) of metastatic spread is characterised by an upregulation of ECM proteins (collagens, fibronectin and laminin, and glycoproteins; aggrecan and thrombospondin) compared with low metastatic risk pUM^7^. Bioinformatic analysis of the secretome data also identified hepatic fibrosis as one of the most differentially upregulated biological processes in HR pUM, suggesting that this is a prerequisite during tumour progression^7^. Indeed, although there are variable hepatic growth patterns in mUM, many exhibit a fibrotic wall surrounding the metastatic tumour nodule^8,9^. This is often associated with a peri-tumoural distribution of lymphocytes, suggesting that this fibrotic wall may hinder their infiltration into the mUM (i.e., creating an “immune cold” tumour nodule)^10^. In this context, the biochemical composition and three-dimensional (3D) structure of the extracellular matrix (ECM) plays a crucial functional and structural role in establishing permissive conditions for local invasion, metastatic spread and response to therapy^11^. However, our overall understanding of ECM composition and how these processes influence tumour progression and tumour cell behaviour in pUM and mUM is limited. The human matrisome is a complex mixture of structural proteins, proteoglycans and glycoproteins that are important not only as cellular scaffolds but also in the provision of molecular cues that can modulate cellular function^12,13^. Recent evidence suggests that both stromal cells and cancer cells can significantly influence matrix structure and composition^14^.

Much pre-clinical cancer research is performed using two-dimensional (2D) cell culture systems that poorly recapitulate TME conditions. Although collagen and Matrigel™ can be used to provide a three-dimensional (3D) matrix in culture, the composition and biochemical properties of these may not be representative of the tumour ECM in vivo. Breakthroughs in the field of tissue engineering include the development of tissue decellularisation methods, which strip a tissue of cells to leave the complex composition of human ECM with preserved tissue architecture^15–17^. This has been used both to understand biochemical and biomechanical aspects as well as provide a biological scaffold for re-cellularisation, thus restoring the function of damaged or dysfunctional tissues. There has been some progress to use similar methods in the study of tumour progression, but these are currently limited, despite their potential to provide information that can be used to develop models that mimic the metastatic niche. Van Tienderen et al., developed an optimized decellularisation technique to characterize the ECM of hepatocellular carcinoma and cholangiocarcinoma, uncovering distinct malignancy-related ECM signatures that would likely be undetected in proteomic analysis of intact tumour material due to protein abundance and resolution^17^. In addition, Xiong et al., demonstrated differences between the abilities of metastatic and non-metastatic breast cancer cells to colonize and grow in decellularised lung matrix^18^.

Our study herein outlines a novel protocol for the decellularisation of pUM tissue that retains its architecture. We examine the protein composition of the decellularised material and identify differences in the ECM signature between high and low-metastatic risk pUM.

## Results

### Decellularisation of pUM tissue for isolation of extracellular matrix scaffold

**Fig. 1:**
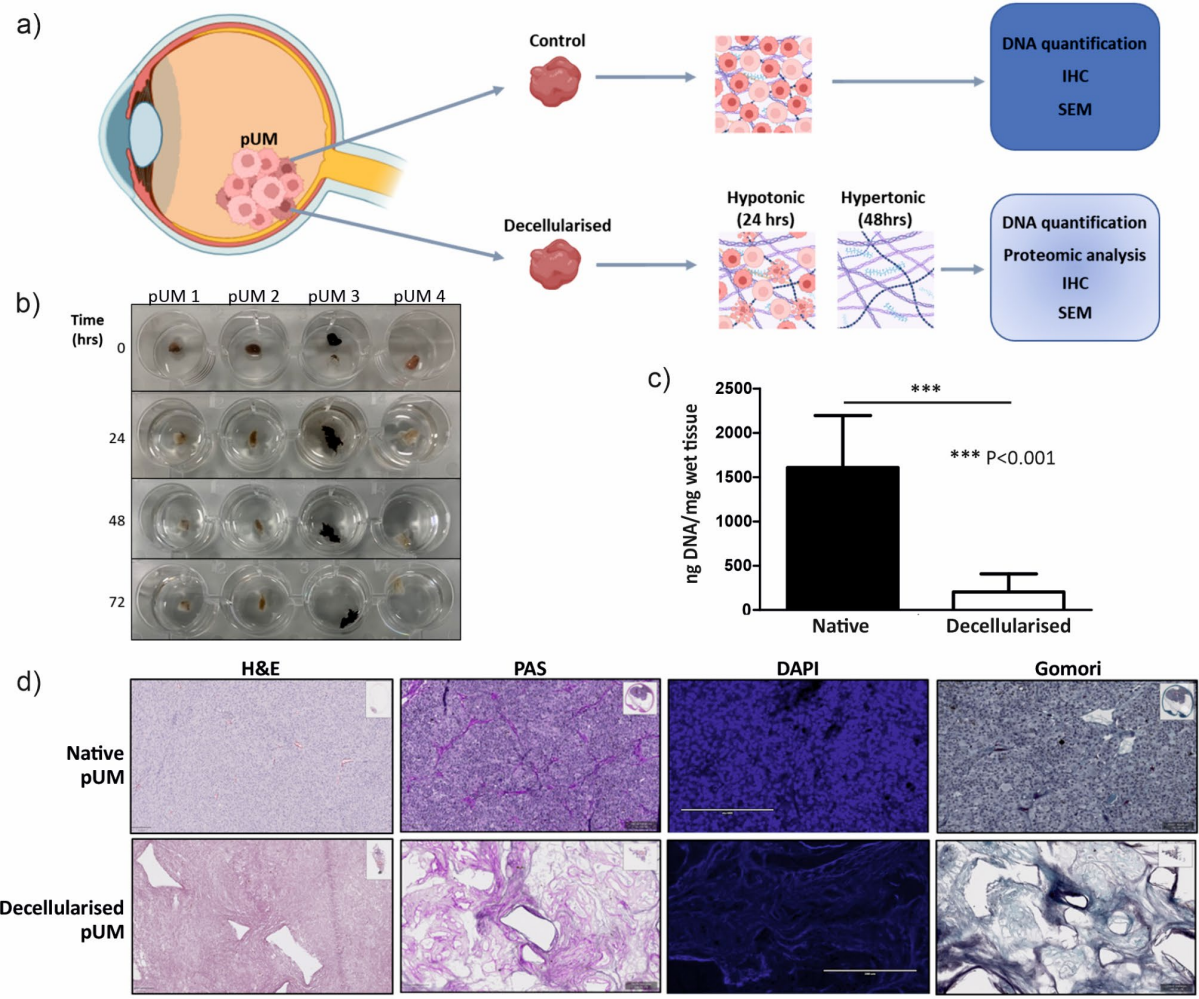
Tumour decellularisation of pUM reveals the ECM scaffold. (**a**) Schematic representation of the decellularisation process and downstream analyses applied to control and decellularised pUM tissue; (**b**) Representative images of four pUM during the decellularisation protocol involving solution changes from hypotonic to hypertonic over 72 h; (**c**) DNA content of decellularised pUM samples compared with matched control tissue to confirm cellular removal during the decellularisation protocol (*n* = 5 per condition); (**d**) Representative Haematoxylin and Eosin (H&E), Periodic Acidic Schiff (PAS), DAPI and Gomori staining of pUM before and after decellularisation demonstrating retention of the ECM scaffold. NB. Part a) created in part with “Biorender.com”.

Primary UM tissue samples of known metastatic risk, with varied pigmentation and structure were decellularised with alternating hypo/hypertonic solutions followed by Triton X-100 incubation (Fig. 1a,b). Quantitative analysis of DNA content (*n* = 5) confirmed successful decellularisation with an average decrease of 87.36% (Fig. 1c) compared with control tissue. In addition, histological analysis of sections of decellularised tissue demonstrated an absence of DAPI stained nuclei when compared with control material confirming successful removal of cellular content (Fig. 1d). The PAS and Gomori stains highlighted the positivity of the connective tissue and collagen fibres often surrounding the tubular ghost-like blood vessels and connective tissue loops, in the acellular tissue architecture (Fig. 1d).

### Proteomic characterisation of decellularised scaffold reveals ECM-associated proteins in HR pUM

**Fig. 2:**
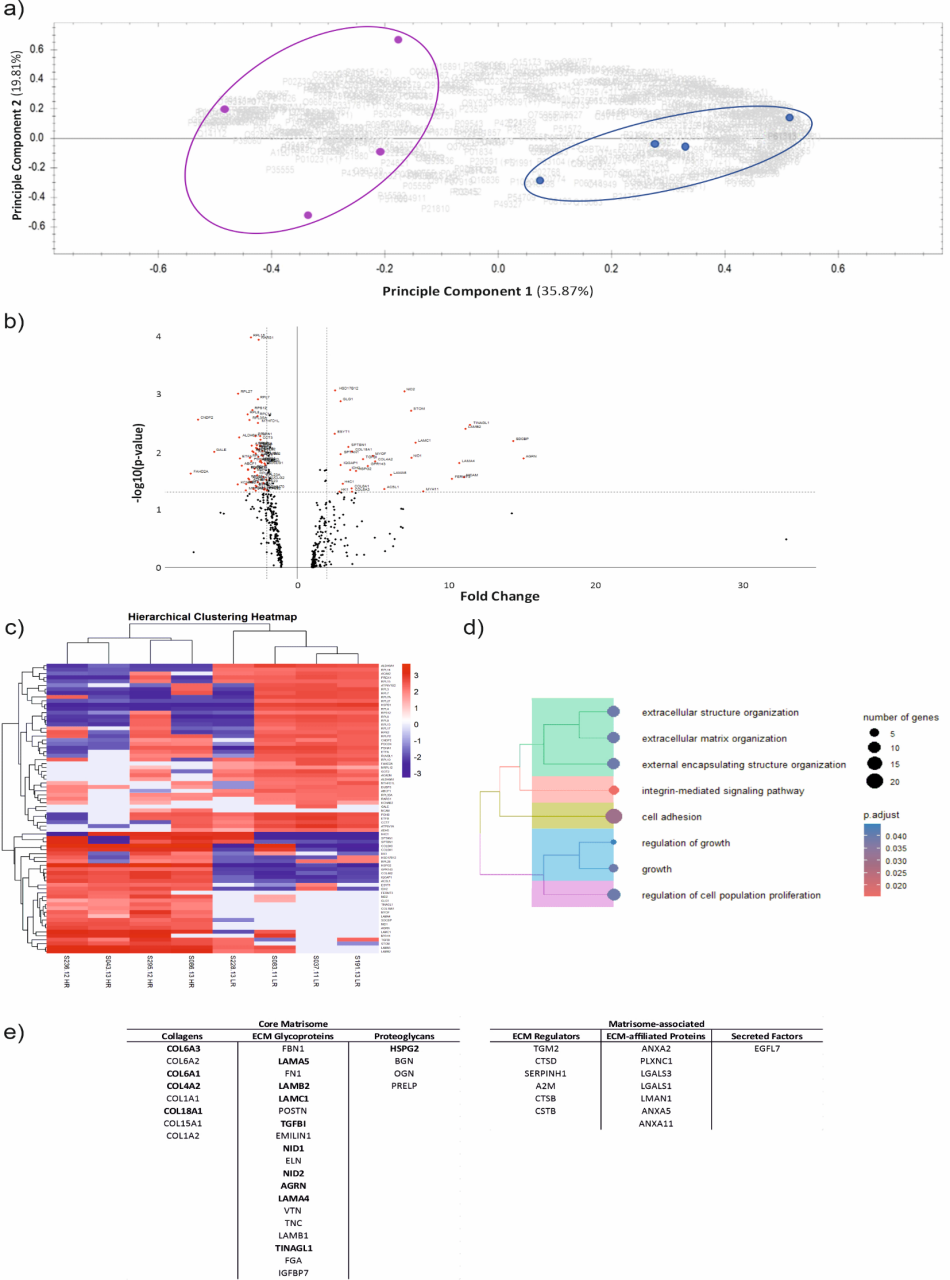
Proteomic analysis of decellularised pUM. (**a**) PCA of 488 proteins identified by LC-MS from decellularised LR-D3 (blue, *n* = 4) and HR-M3 (purple, *n* = 4) pUM samples; (**b**) Volcano plot of differentially expressed proteins with FC ≥ 2, including 93 labelled proteins with *P* < 0.05 (red) Supplementary Table S1 ; (**c**) Hierarchical clustering of pUM samples for the significant 93 proteins (**d**) Tree plot of enriched GO terms for all proteins highlighting ECM pathways; (**e**) 45 proteins identified as ECM-associated using matrisome database 2.0 represented using ECM classifications.

Proteomic analysis identified 488 proteins across all eight pUM samples based on 3 or more unique peptide identifications per protein and an FDR of < 1% (Supplementary Table S1). Principal Component Analysis (PCA) demonstrated separation of HR-M3 (purple) from LR-D3 (blue) pUM based on protein expression (Fig. 2a). Differentially expressed proteins between HR-M3 and LR-D3 pUM groups with a log_2_ fold change ≥ 2, *P* < 0.05 identified 93 proteins (Fig. 2b) and unsupervised hierarchical clustering separated these proteins into HR-M3 and LR-D3 groupings (Fig. 2c). Interestingly sample S228.13, which shows some similarities with HR-M3 UM (Fig. 2c) was noted in the pathology report to have a small focal extraocular melanoma extension at primary diagnosis, which can be associated with an increased risk of metastatic spread. GSEA of all 488 proteins revealed several Gene Ontology (GO) terms relating to extracellular encapsulating structure organisation, cell adhesion and integrin mediated signalling pathways, *P-adjusted* < 0.05 (Fig. 2d).

Using the matrisome database 2.0^19^ to filter the 488 proteins revealed 45 associated with ECM (Supplementary Table S2); 31 core matrisome proteins and 14 matrisome-associated proteins (Fig. 2e). Of the 45 ECM related proteins identified, 34 (76%) were upregulated more than 1.5-fold in the HR-M3 pUM samples (Supplementary Table S3) and 14 with statistical significance, *P* < 0.05 including *NID1*,* COL6A1*,* COL4A2* (proteins in bold Fig. 2e).

### Comparative analysis reveals uniquely expressed ECM proteins

**Fig. 3:**
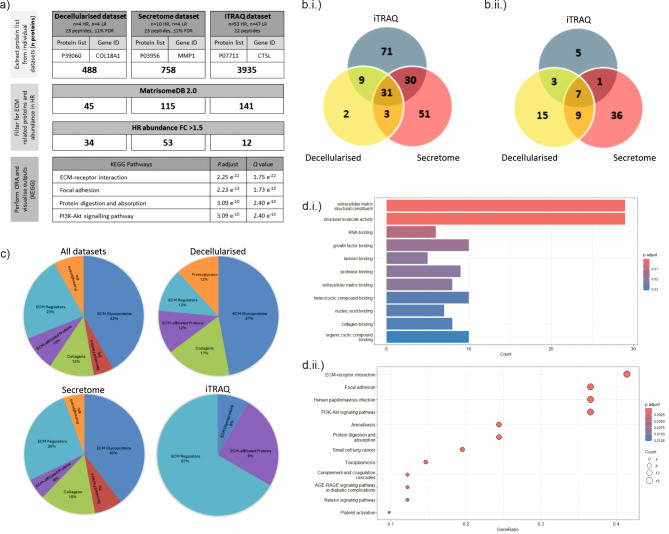
Bioinformatic analysis of in-house datasets (decellularised, secretome, iTRAQ). (**a**) Bioinformatic pipeline of proteomic data processing, and filtering results by HR-M3 metastatic status on the Homo sapiens MatrisomeDB 2.0 background with *P* < 0.05 (in bold are protein hits after each analysis step); (**b**) Venn diagrams reflecting the overlap of proteins from each dataset (i) 197 genes used for ORA, (ii) 76 genes upregulated in HR FC ≥ 1.5; (**c**) Proportion of proteins from 76 genes classified by matrisome nomenclature for combined datasets (All) and separately (decellularised, secretome, iTRAQ) - sub-categories include core matrisome ECM glycoproteins, collagens, proteoglycans and matrisome-associated secreted factors, ECM-affiliated proteins, ECM regulators; & (**d**) ORA pathways showing (i) GO pathways from MF classification, (ii) KEGG pathways^22–24^.

To further evaluate the importance of the identified decellularised proteins, two additional *in-house* and publicly available pUM proteomic datasets - iTRAQ (whole cell)^20^ and secretome (secreted)^7^- were interrogated in a combined analysis approach (Fig. 3a). The datasets differed in the number of proteins identified, ranging from decellularised, *n* = 488, secretome *n* = 758 and iTRAQ *n* = 3935, paralleling the source of each dataset - ECM scaffold to secreted proteins to whole cell. Using all three datasets in downstream analyses removes limitations of each proteomic measurement (with regards to complexity and protein abundance) and increases the power of any resulting biological predictions and pathways. For example, only glycoproteins, secreted factors and collagens were present in the iTRAQ dataset whilst ECM affiliated, ECM regulators and proteoglycans were additionally present in both secretome and decellularised datasets (Fig. 3b).

All datasets were filtered using the matrisome 2.0 database and ECM associated proteins for decellularised, secretome (Supplementary Table S4) and iTRAQ (Supplementary Table S5) were reduced to 45, 115 and 141 proteins, respectively. The combination of the datasets identified 197 unique ECM-related proteins (Fig. 3b.i., Supplementary Table S6). Thirty-one proteins were found in all three datasets including nidogens 1 and 2 (*NID1*,* NID2*), collagens 6A1, A2, A3 (*COL6A1*,* COL6A2*,* COL6A3*), transforming growth factor beta 1 (*TGFB1*), galectin 3 (*LGALS3*) and tenascin-C (*TN-C*) (Supplementary Table S2). Of the 197 proteins, 76 were upregulated in HR-M3 pUM with fold change ≥ 1.5, 64 of those with *P* < 0.05 significance (Fig. 3b.ii., Supplementary Table S7). These 76 proteins were classified by the matrisome subdivisions and categories^21^, and are shown for each dataset separately and combined (Fig. 3c). The seven proteins included in the overlap (FC ≥ 1.5, Fig. 3.b.ii) were collagen 6A3 (*COL6A3*), laminin B2 (*LAMB2*), laminin C1 (*LAMC1*), alpha-2-macroglobulin (A2M), agrin (*AGRN*), cathepsin B (*CTSB*), and collagen 18A1 (*COL18A1*).

### Over representation analysis confirms ECM-receptor involvement in KEGG pathways

Over-representation analysis was used to identify biological functions and pathways enriched in the combined dataset of the 76 proteins upregulated in HR-M3 pUM samples. GO classifications identified 29 cellular components and 10 molecular functions which included ‘structural molecule activity’, and ‘collagen/laminin/integrin binding’ (Fig. 3d.i). Kyoto Encyclopaedia of Genes and Genomes (KEGG^22–24^) revealed 16 pathways with ‘ECM-receptor interaction’ and ‘focal adhesion’ as the top two pathways. Other pathways included ‘PI3K-Akt signalling’, ‘apoptosis’, and ‘complement and coagulation cascade’ (Fig. 3d.ii).

### Validation of proteins identified as upregulated in HR-M3 pUM

#### pUM

**Fig. 4:**
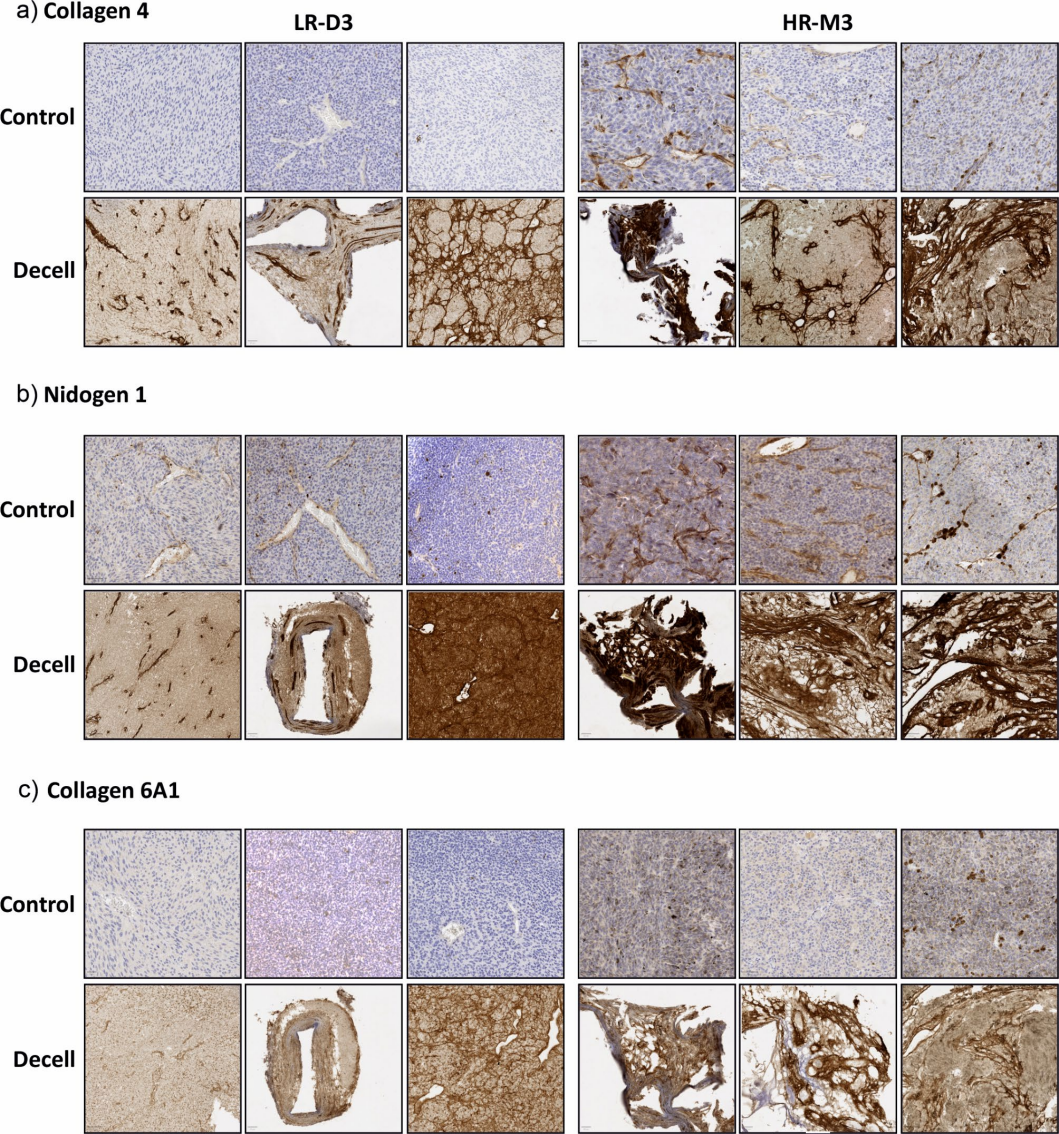
Representative immunohistochemical staining of LR-D3 (*n* = 3) and HR-M3 (*n* = 3) control and decellularised pUM tissues for highly differentially expressed proteins. (**a**) Collagen 4; (**b**) Nidogen 1; (**c**) Collagen 6A1. All scale bars indicate 20/50 µm.

IHC was performed on both control and decellularised formalin-fixed paraffin-embedded pUM tissue sections from three LR-D3 and three HR-M3 pUM samples for collagen 6A1, nidogen 1 and collagen 4. Representative images are shown in Fig. 4. In the control tissue, staining was observed around blood vessels in the normal choroid for all three proteins and in the retina for collagen 4 and nidogen 1, acting as an internal positive control (Supplementary Figure S1). In the tumour regions of the LR-D3 and HR-M3 pUM controls, staining was observed for collagen 4 around a few blood vessels and in the HR-M3 pUM collagen 4 also highlighted looping structures where present. Collagen 6A1 was not detected in any of the LR-D3 pUM controls and was noted to be present around blood vessels and fibrous structures in one of the HR-M3 pUM controls. Nidogen 1 was detected surrounding blood vessels and looping structures where present, in all pUM controls analysed irrespective of metastatic risk, although the levels appeared lower in the LR-D3 pUM most likely due to a lower number of blood vessels and absence of looping structures as would be expected for these cases. Interestingly, in the decellularised tissue, protein expression was more visible for all three proteins in both LR-D3 and HR-M3 pUM samples (Fig. 4). Negative controls showed no staining in the decellularised tissue (Supplementary Figure S2). Although, collagen 6A1, nidogen 1 and collagen 4 staining across the samples varied, the HR-M3 pUM cases appeared to have stronger and more widespread staining than that present in the LR-D3 samples. Moreover, the patterns of staining in the decellularised material were consistent with ghost-like vascular structures^25,26^ and the ECM scaffold as also shown with SEM in Fig. 6. Additional staining with CD34 in 2 decellularised cases (S203.21 LR-D3, S295.12 h-M3) confirmed the presence of vascular structures in concordance with PAS staining (Supplementary Figure S3).

**Fig. 5:**
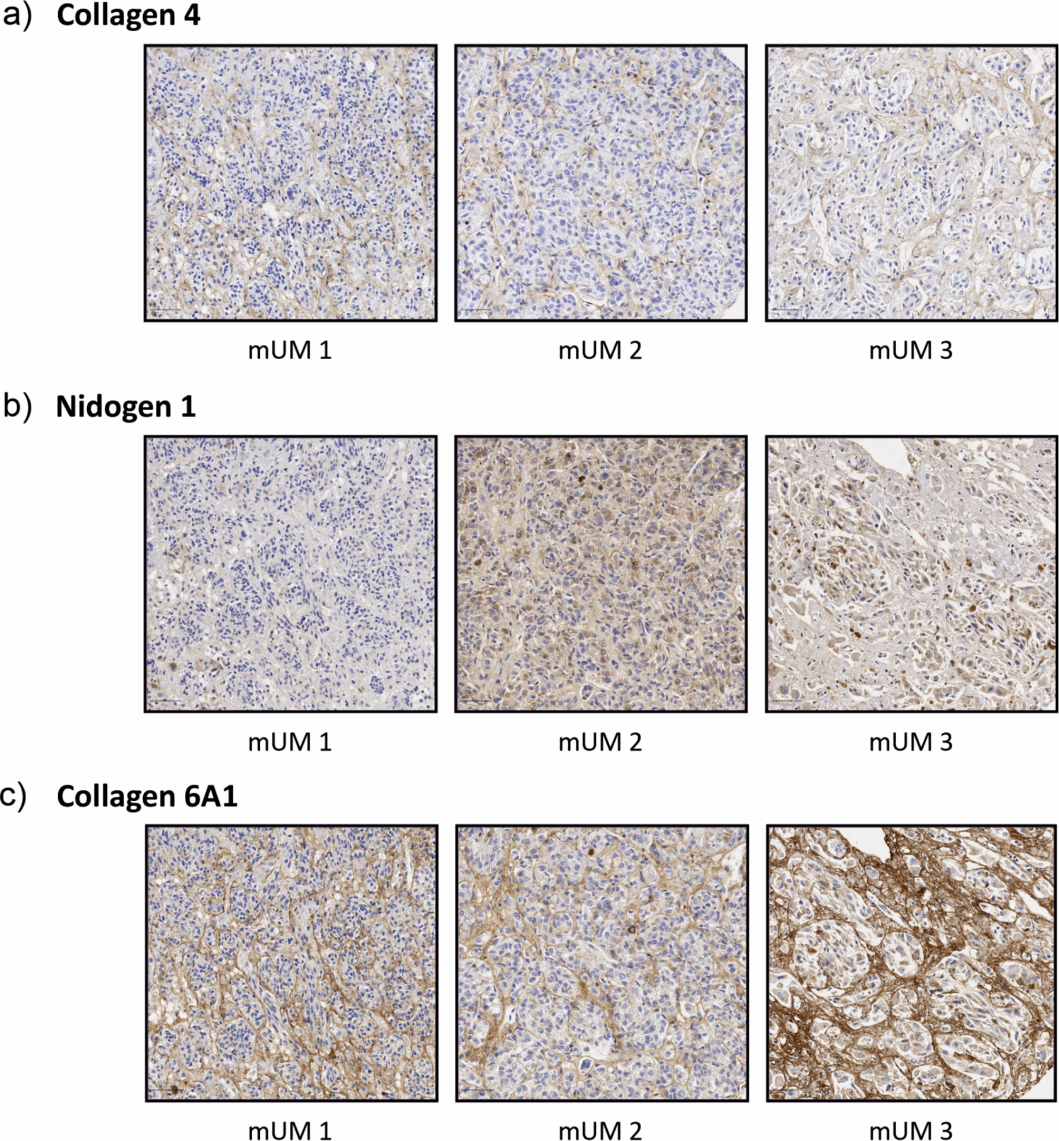
Representative immunohistochemical staining of mUM tissues for highly differentially expressed proteins (*n* = 3). (**a**) Collagen 4; (**b**) Nidogen 1; (**c**) Collagen 6A1. All scale bars indicate 50 μm.

**Fig. 6:**
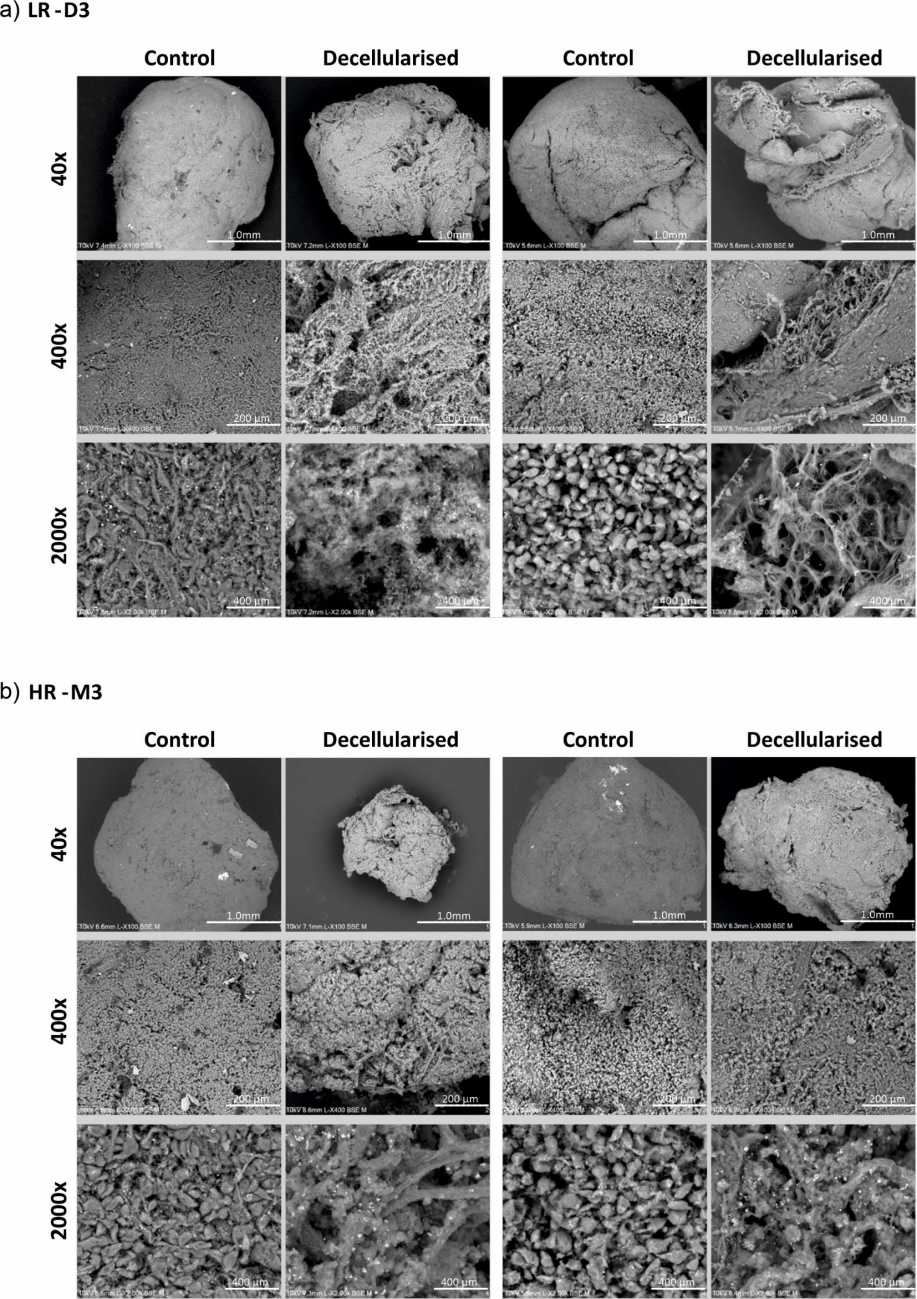
Representative SEM images of LR-D3 and HR-M3 control and decellularised tissues (*n* = 2). (**a**) LR-D3 matching control and decellularised tissues at 40x, 400x and 2000x magnification; (**b**) HR-M3 matching control and decellularised tissues at 40x, 400x and 2000x magnification.

#### mUM

Tissue from three cases of non-decellularised hepatic metastatic UM were also stained with collagen 4, nidogen 1 and collagen 6A1 (Fig. 5). The staining was more visible and more widespread than seen in the control pUM samples although staining patterns were similar, highlighting the vascular structures, connective tissue loops and collagen-rich ECM lattice.

### Scaffold architecture analysis of decellularised tissue by SEM

SEM was employed to provide high resolution, 3D images of the structure of pUM samples, both control and decellularised, allowing more in depth topographical, morphological, and compositional detail to be seen (Fig. 6). Both control LR-D3 and HR-M3 pUM tissues revealed densely packed cellular structures with cell shape and size easily observed at 2000x magnification. Epithelioid and spindle cell morphology was seen at the highest magnifications and matched the pathological characteristics. All cell types appeared interconnected and in close contact and it was difficult to distinguish any clear fibre structures within control tissues. SEM of the decellularised tissue revealed the effective removal of cellular matter from all tissues and clear fibrillar structures could be seen (Fig. 6).

In terms of structure, decellularisation of HR-M3 pUM cases revealed ECM that appeared more compact and less porous (Fig. 6b) than that observed in LR-D3 pUM (Fig. 6a), possibly indicating remodelling of the interstitial matrix.

## Discussion

Our study represents the first comparative analysis of the ECM-specific proteome in pUM of differing metastatic risk. We have: (1) identified a matrisome signature of core ECM and ECM-associated proteins upregulated in HR-M3 pUM when compared with LR-D3 pUM that may play a functional role in UM progression, which could be targeted and/or serve as biomarkers; and (2) developed a method for the decellularisation of pUM tissue that retains much of the biophysical scaffold that may serve as a platform for re-cellularisation and/or inform the creation of bioengineered matrices. In particular, whole tumour tissue decellularisation methods generated a global ECM secreted by both tumour and stromal cells, which removes the limitations of scaffolds created in vitro by or based solely on stromal cells, such as fibroblasts.

The number of articles describing the importance of the ECM in tumours and their progression has more than doubled in the last 10 years, although most works have been undertaken on large transcriptomic datasets^27,28^, or in mouse models^29–31^. Relatively few studies have been undertaken in human tumour tissue^32^ and, indeed, this is the first study to use proteomic analysis of HR-M3 pUM and LR-D3 pUM to identify a matrisome profile associated with metastatic risk. In addition, histological, immunohistochemical, and ultrastructural evaluations of decellularised pUM confirmed the preservation of native tissue architecture and major ECM components including laminin, collagens, and glycosaminoglycans (GAG), such as heparin sulfate proteoglycan 2; GAG preservation is crucial to maintain the biological activity of the decellularised scaffolds^33^.

Perturbed collagen architectures are often observed surrounding tumours likely due to cellular remodelling of the ECM associated with tumour stiffness. Lysyl oxidases catalyse cross-linking reactions between collagen and elastin to regulate ECM formation, development, maturation, remodelling and thus stiffness^34^. Although ECM stiffness was not examined in this study, LOXL2 and LOXL4 were upregulated in HR-M3 pUM. Kaluz et al., analysed The Cancer Genome Atlas (TCGA) data and reported *Col6A1* and *Col6A2* as the most abundantly expressed collagen genes in pUM^35^, which is consistent with the abundance of collagens 6A1, A2 and A3 proteins in HR-M3 pUM and the high expression of this collagen in mUM in our study. Similarly, collagen 6 was detected by Daniels et al., in choroidal melanoma by IHC and not in the normal choroid, reflecting the remodelling potential of ECM by tumour cells^36^. Of note, Li et al. who examined the 33 TCGA cancers analysed in detail, reported collagens 6A1, A2 and A3 to be upregulated in several malignancies associated with a TGFβ prominent immune profile and a poor outcome^37^. Their study also reported collagens 6A1, A2 and A3 as potential treatment targets, due to their associations with chemotherapeutic sensitivity/resistance^37^. This could provide a unique opportunity for drug targeting in UM based on collagen 6 expression, exploring the use of antifibrotic drugs. Older UM studies implicated collagen 6 in ECM remodelling and the authors hypothesized that collagen 6 and hyaluron were precursors of vascular networks, e.g., closed ‘vascular connective tissue loops’ that are often seen in poor prognosis UM^36^. Indeed, these closed connective tissue loops were present in all HR-M3 samples used in our study for proteomic analysis; however, the small sample size examined does not allow any further correlations with these features in this study. Kaluz et al.,^35^ also reported upregulation of the hypoxia regulated genes *P4HA1* and *P4HA2* in UM patients with metastatic disease, consistent with upregulation of P4HA1 protein in HR-M3 pUM in our study; these genes in turn regulate collagen maturation and deposition. Other collagens shown to be upregulated in the HR-M3 pUM datasets have also been associated with poor prognoses in several tumour types. Collagen 4 is secreted by pancreatic cancer cells, with circulating and stromal collagen 4 associated with poor survival^38^. Type 4 collagens form a supramolecular structure involved in the basement membrane that influences adhesion and migration of epithelial cells^39^. In our study, collagen 4A2 was upregulated in HR-M3 pUM and a pan-collagen 4 antibody identified collagen fibres in both HR-M3 pUM and mUM tissues. Collagen 4 anchors the tumour cells, and a study in UM showed increased cell adhesion with a collagen 4 matrix and invasive UM cells^40^, potentially stimulating growth and migration^38^.

Metastases occur in approximately 50% of UM patients via the bloodstream predominantly to the liver^41^. In a prior study, ECM networks were identified in collagen I gel matrices remodelled by isolated pUM cells; and implicated the interaction of laminins and metalloproteinases (MMPs) in the degradation and formation of these networks^42^. Within our datasets collagen 1A1 was upregulated in HR-M3 pUM as was MMP1 and ADAM10. ADAM10 has previously been reported as highly expressed in UM cell lines with gene silencing resulting in reduced invasiveness of 92.1 μm cells^43^.

Consistent with analyses in other tumour types, we identified Tenascin-C in high abundance and upregulated in HR-M3 pUM. Tenascin-C has widespread protein distribution in embryonic tissues, where it is found surrounding motile cells^44^, whilst in adult tissues it has a more restricted distribution associated with inflammation^45^ and stem cell niches^46^, perhaps linking this ECM protein with a more dedifferentiated phenotype of HR-M3 pUM. Recent studies using a pancreatic neuroendocrine mouse model showed knocking out Tenascin-C decreased angiogenesis thereby suggesting this molecule as an angiogenic modulator^47^, consistent with increased vascular loops in HR-M3 pUM. Tenascin-C has recently been proposed as a prognostic biomarker of increased UM mortality^48^. Several agents either targeting Tenascin-C directly or combination therapies with Tenascin-C antibodies are in clinical trials in other cancers^49^.

Metastatic UM is refractory to most immune checkpoint inhibitors and other immunotherapy modalities^50^. Evidence suggests that soluble galectins released in the TME bind specific glycoproteins and glycolipids exposed on the plasma membrane of tumour infiltrating lymphocytes (TIL) modulating their function thus contributing to tumour immune escape^51^. We previously reported upregulation of galectin 3 in hepatic mUM^52^, and both galectin 3 and galectin 7 were identified as part of the HR-M3 upregulated matrisome in this study suggesting the relevance of galectin targeting therapies in mUM.

Several other factors detected as upregulated in HR-M3 pUM in this study have previously been associated with tumour progression and metastasis in UM, including growth differentiating factor 15 (*GDF-15*), thrombospondin 2 (*THBS2*), and cysteine-rich angiogenic inducer 61 (*CYR61*)^53–55^.

Our SEM studies of pUM decellularised ECM are novel; SEM revealed compacted tumour cells in non-decellularised control tissue with few ECM fibres, whilst intact fibres forming complex networks are more demonstrable in the decellularised tissue, both for HR-M3 and LR-D3 tissue.

The current study provides proteomic and structural characterisation of the ECM of pUM; however, with metastatic disease remaining the main challenge in UM, characterisation of mUM is vital to better understand the role of ECM in metastases. This study has successfully established a ‘proof of concept’ methodology for effective decellularisation of pUM with the aim of utilising this technique in metastatic UM tissue, which is a limited resource, due to the rare nature of this disease and the small number of liver resections performed per year. Despite this, expression of Collagen 4, Collagen 6A1 and Nidogen 1 proteins by IHC in mUM tissue did show expression levels resembling pUM HR-M3 tissue, and suggested ECM morphology similarities with pUM. Previous studies have also reported morphological and ECM similarities between primary and metastatic UM^8,9^, with the latter manipulating the liver parenchyma to create distinct hepatic growth patterns that support its growth^8,9,56,57^. Such ‘mirror-like’ reconstruction of tumour morphology and supporting matrisome has been seen in other malignancies, e.g. metastatic colon cancer and breast cancer^31,58^.

## Conclusions

Our study provides a novel approach to studying the ECM protein composition of pUM and offers unique insights into metastatic risk-specific protein profiles. These data will be linked to mUM ECM protein composition and stiffness in future studies, to help define proteins involved in guiding tumour cells to the hepatic premetastatic niche. By combining this with the bioinformatic analyses and GO and KEGG pathways already revealed in this study, which highlight ECM receptor interaction and PI3K/AKT signalling, it is now crucial to dissect the molecular mechanisms regulated by these proteins. Critically, the generation of 3D biomaterial scaffolds resulting from these analyses will provide new insights into cellular behaviour, and importantly provide a platform to test novel therapeutics targeting the ECM.

## Methods

### Clinical samples

This study conformed to the principles of the Declaration of Helsinki, and all procedures and methods relating to the human tissue used were approved by the Health Research Authority under the REC Ref 11/NW/0568. All samples and pseudo-anonymized data including clinical, histopathological (including nuclear BAP1 (nBAP1) protein expression) and genetic information (chromosome 3 and 8q copy number) were provided by the Ocular Oncology Biobank (REC ref 21/NW/0139). All patients had provided informed consent for the use of their samples and data in research. All samples were snap frozen following isolation and stored long-term at -80^o^C or were available as formalin fixed paraffin embedded tissue.

DNA quantification was undertaken in initial protocol optimisation on five anonymized primary UM (pUM) samples not included in any downstream processes to confirm acellular material.

#### Proteomic samples

Four pUM samples classified using chromosome 3 status as high metastatic risk monosomy 3 (HR-M3) and four classified as low metastatic risk disomy 3 (LR-D3), where the patients had died of metastasis within 5 years of follow-up or were still alive respectively, were selected for decellularisation and proteomic analysis.

#### Immunohistochemistry (IHC) and scanning electron microscopy (SEM) samples

Six pUM samples of known metastatic risk were also selected for decellularisation, IHC and SEM analysis.

Patient data for pUM samples used in proteomic-, IHC- and SEM analyses are detailed in Table 1.

#### mUM IHC samples

mUM tissue samples (*n* = 3) were used for IHC staining with selected antibodies.

**Table 1:** Patient demographics and clinicopathological features.

Sample No.	Age at PM (yrs)	Gender	LBD (mm)	UH (mm)	CBI	EOE	Loops	Epithelioid cells	Mitotic Count per 40 HPF	Chr3 status	Chr8q status	nBAP1	Mets risk	Survival	Prot	IHC & Hist	SEM
S228.13	47	M	14.6	6	No	Yes	No	No	2	N	G	POS	LR	Alive	Yes		
S191.13	75	M	15.8	11.1	No	No	No	No	5	N	G	POS	LR	Alive	Yes		
S043.13	87	F	18.3	12.6	Yes	No	Yes	Yes	2	L	G	NEG	HR	Dead	Yes		
S086.13	56	M	14	9	No	No	Yes	Yes	5	L	G	NEG	HR	Dead	Yes		
S083.11	63	M	13.5	8.4	No	No	No	No	10	N	N	POS	LR	Alive	Yes	Yes	Yes
S037.11	74	F	19	11.3	No	No	No	No	5	N	N	POS	LR	Alive	Yes	Yes	Yes
S295.12	60	F	12.7	13.2	No	No	Yes	Yes	9	L	G	NEG	HR	Dead	Yes	Yes	Yes
S236.12	71	M	15	18.3	Yes	No	Yes	Yes	18	L	G	NEG	HR	Dead	Yes	Yes	Yes
S203.21	42	M	16.1	15.6	Yes	No	Yes	No	6	N		POS	LR	Alive^*^		Yes	Yes
S200.21	30	M	19.1	9.5	Yes	No	No	Yes	4	L	G	NEG	HR	Alive^*^		Yes	Yes

*PM* primary management, *M* Male, *F* Female, *LBD* largest basal diameter, *UH* ultrasound height, *CBI* ciliary body involvement, *EOE* extraocular extension, *Pos* positive, *Neg* negative, *HR* high risk, *LR* low risk, *N* normal, *L* loss, *G* gain, Survival with * indicates follow-up <5 years, *Prot* proteomic analysis, *Hist* histological analysis.

### Decellularisation procedure

pUM samples selected had sufficient tissue available to yield two samples between 15 and 35 mg in weight, to provide both ‘control’ (non-decellularised) and ‘decellularised’ specimens. Tissue samples were decellularised in individual wells of a 12-well plate over a 72-hour period. In brief, samples were incubated in 1 ml hypotonic solution (10mM Tris-HCl, 5mM EDTA, pH8.0) for 24 h on a shaking platform at room temperature (RT). The solution was then removed and the tissue washed for 15 min in 1 ml phosphate buffered saline (PBS) before the addition of 1 ml of hypertonic solution (50 mM Tris-HCl, 0.5 M NaCl, 10 mM EDTA, pH8.0) for a further 48 h at RT. Tissue samples were then washed for 15 min in 2 ml PBS before incubation in 0.5% TX100 in dH_2_O for 3 h and a final incubation in dH_2_O for 1 h. Following decellularisation, the tissue pieces were either snap frozen and stored at -80 °C prior to proteomic analyses, fixed in 10% neutral buffered formalin (NBF) for processing and paraffin embedding, fixed in glutaraldehyde/paraformaldehyde (PFA) solution for SEM, or underwent DNA extraction.

### DNA quantification

The efficiency of the tissue decellularisation process was initially confirmed by assessing DNA content across five pUM samples. DNA extraction was performed with the Qiagen Blood and Tissue DNA extraction kit according to manufacturer’s instructions with an overnight extended proteinase K incubation. DNA was quantified using the NanoDrop spectrophotometer (ThermoFisher, UK). Data are mean ± SD, compared using unpaired parametric student’s t-test, *P* < 0.05 considered significant.

### Proteomic sample preparation

#### Lysis and protein estimation

Each sample was weighed and nine times the weight in volume of complete EDTA free protease inhibitor cocktail in 25 mM Ammonium Bicarbonate **(**AmBic) was added (e.g., 10 µg of sample, 90 µL of Lysis buffer). Protein concentration was estimated for each sample using a Bradford assay.

#### In-solution digestion

 50 µg total protein from each sample was denatured in 1% Rapigest™ solution heated to 80˚C for 10 min. Cysteine reduction was performed with dithiothreitol in 25 mM Ambic incubated at 60˚C for 10 min. Subsequent alkylation was performed by adding iodoacetamide in 25 mM Ambic and incubating for 30 min in the dark. Digestion was performed with 0.2 µg/µL trypsin (50:1 ratio of sample: trypsin) at 37˚C for 16 h on an orbital shaker. Rapigest™ inactivation was achieved by adding trifluoroacetic acid, ensuring a pH of 2 or less. Finally, samples were incubated at 37˚C for 45 min before spinning at 13,000 x *g* for 15 min at 7 ˚C.

#### LC-MS analysis

Samples were analysed with an Ultimate 3000 RSLC™ nano-system (Thermo Scientific) coupled to a Q Exactive Quadrupole-Orbitrap™ mass spectrometer (Thermo Scientific). The data-dependent program used for data acquisition consisted of a 70,000-resolution full-scan MS scan in the orbitrap. All samples were analysed in random order.

#### Data analysis

Data were searched using Proteome Discoverer (v 2.4) and the Mascot search engine (v 2.8) against the UniProt database of human reviewed proteins. Fixed cysteine carbamidomethylation and variable modification of methionine oxidation were specified and limited to 1 missed cleavage. Data was processed using Progenesis. Label free quantitation was performed on the top 3 unique and razor peptides. Proteins identified with less than 3 unique/razor peptides and greater than 1% FDR were filtered out.

### Bioinformatic analyses

Bioinformatic analyses were performed with R 4.3.2 (cran.r-project.org) on the decellularised dataset generated above and two additional independent proteomic datasets, including secretome protein data from pUM specimens (*n* = 10 h; *n* = 4 LR)^7^ and isobaric tag for relative and absolute quantification (iTRAQ) labelled pUM protein data (*n* = 53 h; *n* = 47 LR)^20^. Proteins in each dataset were interrogated using the following criteria: decellularised dataset - ≥3 unique peptides with a 1% False Discovery Rate (FDR); secretome dataset - ≥3 unique peptides with a 1% FDR; and iTRAQ dataset – ≥2 unique peptides relative to a pooled non-involved choroid control from UM eyes. For each dataset proteins were converted to the gene name and gene entrez ID numbers were obtained using the maplds function from the org.Hs.eg.db R package. Initial analysis was performed on the decellularised dataset al.one using gene set enrichment analysis (GSEA) R package “clusterProfiler” version 4.10.0 function gseGO. Subsequent combined dataset analyses used proteins that were only present in the publicly available *Homo sapiens* Matrisome database 2.0 (matrisomedb.org accessed date: 11 December 2023^19^). Differentially expressed proteins upregulated in HR-M3 pUM defined by a fold change (FC) ≥ 1.5 for both decellularised and secretome datasets, and ≥ 1 std dev from the mean HR: LR linear ratio for the iTRAQ dataset, were taken forward. Over Representation Analysis (ORA) was performed using the R package “clusterProfiler” version 4.10.0^59^. GO analysis was performed using the function EnrichGO with background gene list set to the matrisome database 2.0 as above. KEGG analysis was performed using EnrichKEGG function with background set to ‘*Homo sapiens’*.

### Histology and immunohistochemistry

Sections of non-decellularised ‘control’ and decellularised pUM tissue were stained with; Haematoxylin and Eosin (H&E) to highlight tissue architecture, Periodic Acid Schiff (PAS) to highlight the basement membrane, Gomori trichrome to detect collagen, and 4′,6-diamidino-2-phenylindole (DAPI) to identify cell nuclei.

Immunohistochemical detection of key ECM proteins identified following bioinformatic analyses was undertaken in sections of control and decellularised pUM and mUM tissue using the Leica Bond RXm and Bond Polymer Refine detection kit according to the manufacturer’s instructions. Goat antibodies required removal of the post primary step and addition of a rabbit anti goat IgG as a linker for the horse radish peroxidase enzyme. Antibodies and conditions for antigen retrieval are provided in Table 2.

**Table 2:** Antibodies and conditions for IHC.

Antibody	Antigen	Species	Antigen Retrieval	Concentration (µg/ml)
Sigma C1926	Col4	Mouse	High pH	16
Merck HPA029401	Col6A1	Rabbit	High pH	1
BioTechne AF2570	Nid1	Goat	High pH	4

### Scanning electron microscopy (SEM)

Matched control and decellularised samples were fixed with glutaraldehyde/paraformaldehyde (PFA) fixative solution (0.5 ml 2.5% glutaraldehyde, 1.25 ml 4% PFA, 0.5 ml 1 x phosphate buffered solution (PBS), 2.75 ml dH_2_O) overnight at 4^o^C. Samples were washed with PBS x3, placing on a test tube rotator for 3 min in-between washes. Samples were placed in osmium tetroxide (OsO_4_) stain (2% working solution, filtered) overnight. Samples were then washed 5x in dH_2_O for 5 min on the rotator and dehydrated using graded ethanol (33-, 50-, 70-, 90-, 100%) for 10 min again on the rotator. A second and final 100% ethanol wash step was performed, and the sample cooled to approximately 5^o^C using critical point drying to replace EtOH with liquid CO_2_. Samples were then heated until pressure reached 90 Bar, upon which the pressure was allowed to release slowly overnight. Inert samples were placed on carbon stubs and coated with 5 µn gold plating and viewed on FEG-SEM using TM4000 software.

## Electronic supplementary material

Below is the link to the electronic supplementary material.

Supplementary Material 1

Supplementary Material 2

Supplementary Material 3

Supplementary Material 4

Supplementary Material 5

Supplementary Material 6

Supplementary Material 7

Supplementary Material 8

## Data Availability

The processed data required to reproduce the decellularised proteomic findings and secretome/iTRAQ matrisome proteins can be found in Supplementary Tables S1, S4 & S5 included within this manuscript or in our previous publication respectively (https://www.ncbi.nlm.nih.gov/pmc/articles/PMC8307952/pdf/cancers-13-03520.pdf).

## Abbreviations

UM: Uveal melanoma
TME: Tumour microenvironment
3D/2D: 3/2–dimensional
ECM: Extracellular matrix
pUM: Primary uveal melanoma
LR: D3–low metastatic risk disomy 3
HR: M3–high metastatic risk monosomy 3
mUM: Metastatic uveal melanoma
PCA: Principal component analysis
iTRAQ: Isobaric tag for relative and absolute quantification
GO: Gene ontology
KEGG: Kyoto Encyclopaedia of Genes and Genomes
ORA: Over representation analysis
GSEA: Gene set enrichment analysis
IHC: Immunohistochemistry
SEM: Scanning electron microscopy
