# Supplementary material for: Revealing the structural microenvironment of high metastatic risk uveal melanomas following decellularisation
Source: Sci Rep. 2024 Nov 5;14:26811. doi: 10.1038/s41598-024-78171-2 (PMC11538295; doi:10.1038/s41598-024-78171-2)
Supplement: Supplementary file 8 — Supplementary Material 8 [file 41598_2024_78171_MOESM8_ESM.docx]

**Revealing the structural microenvironment of high metastatic risk uveal melanomas following decellularisation.**

Karen Aughton,^1*^ Joshua Hattersley,^1^ Sarah E Coupland ^1,2^ and Helen Kalirai.^1,2^

^1^Liverpool Ocular Oncology Research Group, University of Liverpool, Department of Eye and Vision Science, Institute of Life Course and Medical Science, University of Liverpool, UK.

^2^Liverpool Clinical Laboratories, Liverpool University Hospital Foundation Trust, Liverpool, UK.

**Supplementary Material**


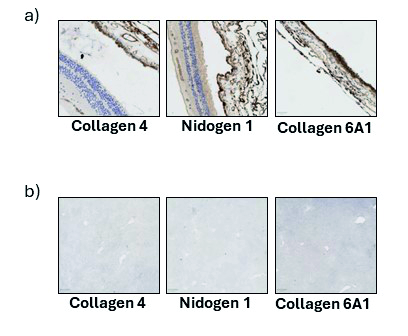


**Supplementary Figure S1:** Positive internal controls for collagen 4 (choroid and retina), nidogen 1 (choroid and retina), and collagen 6A1 (choroid only). Positive staining is seen around the blood vessels. Scale bar indicates 50 µm.


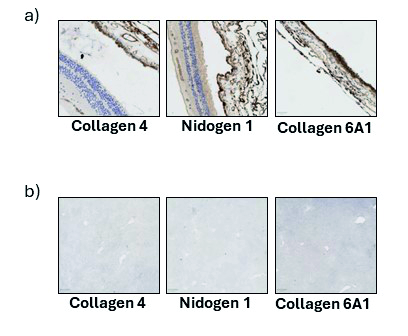


**Supplementary Figure S2:** Negative controls for collagen 4 (mouse IgG1 16 µg/ml), nidogen 1 (antibody diluent), and collagen 6A1 (rabbit IgG 4 µg/ml) in decellularised tissue showing no staining. Scale bar indicates 50 µm.


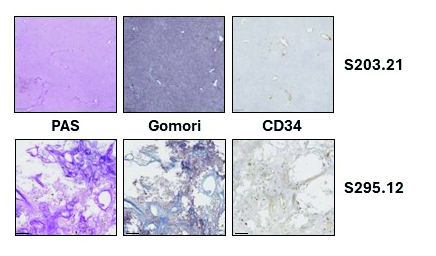


**Supplementary Figure S3:** Decellularised tissue from LR-D3 (S203.21) and HR-M3 (S295.12) pUM stained with Periodic Acid Schiff, Gomori and CD34 highlighting vasculature in the tissues. Scale bar indicates 50 µm.

**Supplementary Table S1:** 488 proteins identified in decellularised pUM.

**Supplementary Table S2:** 45 Matrisome database filtered ECM proteins from the decellularised dataset.

**Supplementary Table S3:** 34 matrisome proteins upregulated in HR pUM with FC ≥1.5 from the decellularised dataset.

**Supplementary Table S4:** 115 matrisome proteins identified from pUM secretome data.

**Supplementary Table S5:** 141 matrisome proteins identified from pUM by iTRAQ.

**Supplementary Table S6:** Gene names of 197 matrisome proteins identified from decellularised, secretome and iTRAQ datasets.

**Supplementary Table S7:** 76 matrisome proteins found in all datasets upregulated in HR with FC ≥1.5.
